# Incorporating external evidence in trial-based cost-effectiveness analyses: the use of resampling methods

**DOI:** 10.1186/1745-6215-15-201

**Published:** 2014-06-03

**Authors:** Mohsen Sadatsafavi, Carlo Marra, Shawn Aaron, Stirling Bryan

**Affiliations:** 1 Institute for Heart and Lung Health, Faculty of Medicine, University of British Columbia, 317 – 2194 Health Sciences Mall (Woodward Instructional Resource Centre), Vancouver, Canada V6T 1Z3; 2 Collaboration for Outcomes Research and Evaluation, Faculty of Pharmaceutical Sciences, University of British Columbia, 2405 Wesbrook Mall, Vancouver, Canada V6T 1Z3; 3 Centre for Clinical Epidemiology and Evaluation, Vancouver Coastal Health Institute, University of British Columbia, 7th Floor, 828 West 10th Avenue, Research Pavilion, Vancouver, Canada V5Z 1M9; 4 Faculty of Health Sciences, University of Ottawa, 451, Smyth Road, Ottawa, Canada K1H 8M5; 5 School of Public and Population Health, University of British Columbia, 2206 East Mall, Vancouver, Canada V6T 1Z3

**Keywords:** Cost-benefit analysis, Bayes theorem, Clinical trial, Statistics-nonparametric

## Abstract

**Background:**

Cost-effectiveness analyses (CEAs) that use patient-specific data from a randomized controlled trial (RCT) are popular, yet such CEAs are criticized because they neglect to incorporate evidence external to the trial. A popular method for quantifying uncertainty in a RCT-based CEA is the bootstrap. The objective of the present study was to further expand the bootstrap method of RCT-based CEA for the incorporation of external evidence.

**Methods:**

We utilize the Bayesian interpretation of the bootstrap and derive the distribution for the cost and effectiveness outcomes after observing the current RCT data and the external evidence. We propose simple modifications of the bootstrap for sampling from such posterior distributions.

**Results:**

In a proof-of-concept case study, we use data from a clinical trial and incorporate external evidence on the effect size of treatments to illustrate the method in action. Compared to the parametric models of evidence synthesis, the proposed approach requires fewer distributional assumptions, does not require explicit modeling of the relation between external evidence and outcomes of interest, and is generally easier to implement. A drawback of this approach is potential computational inefficiency compared to the parametric Bayesian methods.

**Conclusions:**

The bootstrap method of RCT-based CEA can be extended to incorporate external evidence, while preserving its appealing features such as no requirement for parametric modeling of cost and effectiveness outcomes.

## Background

Randomized controlled trials (RCTs), especially ‘pragmatic’ RCTs that measure the effectiveness of interventions in realistic settings, are an attractive opportunity to provide information on cost-effectiveness [1]. In the context of such a RCT, many aspects of treatment from clinical outcomes to adverse events and costs are measured at the individual level, which can be used to formulate an efficient policy based on cost-effectiveness principles. A growing number of trials incorporate economic endpoints at the design stage and there are established guidelines for conducting a cost-effectiveness analysis (CEA) alongside a RCT [2,3].

The statistic of interest in a CEA is the incremental cost effectiveness ratio (ICER), which is defined as the difference in cost (∆*C*) between two competing treatments over the difference in their health outcome (effectiveness) (∆*E*). With patient-specific cost and health outcomes at hand, estimating the population value of the ICER from an observed sample becomes a classical statistical inference problem. However, given the awkward statistical properties of cost data and some health outcomes such as quality-adjusted life years (QALYs), and issues around parametric inference on ratio statistics, many investigators choose resampling methods for quantifying the sampling variation around costs, health outcomes, and the ICER [4]. In parallel-arm RCTs, this can be performed by obtaining a bootstrap sample within each arm of the trial and calculating the mean cost and effectiveness within each arm from the bootstrap sample; repeating this step many times provides a random sample from the joint distribution of arm-specific cost and effectiveness outcomes. This sample can then be used to make inference on (such as calculate the confidence or credible interval for) the ICER [5].

Recently, such a framework for evaluating the cost and outcomes of health technologies has received some criticism [6-8]. Specifically, critics argue that making decisions on the cost-effectiveness of competing treatments should be based on all the available evidence, not just those obtained from a single RCT [8]. In this context, evidence synthesis is the practice of combining multiple sources of evidence (from other RCTs, expert opinion, and case histories) in informing the treatment decision, a task that is quantitatively performed using the Bayes’ rule [9].

A conventional analysis of a clinical trial often involves making inference primarily on the effect size and secondarily on other aspects of treatment such as safety or compliance. These measures are conceptually distinct enough to be analyzed and reported separately and trialists have a full arsenal of standard statistical methods at their grasp for such analyses. Evidence synthesis is often conducted separately, usually through quantitative meta-analysis, after the results of several studies are available. An economist, on the other hand, does not have the luxury of dissecting RCT results into different components as cost-effectiveness is a function of all aspects of an intervention. As such, evidence external to the trial on any aspect of treatment has bearings on the results of the CEA. In addition, when a RCT is used as a vehicle for the CEA the incorporation of external evidence must be part of the analysis. Results of a CEA have direct policy implications and the economist cannot defer evidence synthesis to any subsequent stage [8].

For trial-based CEAs, if external evidence on cost or effectiveness is available then the investigator can use standard parametric Bayesian methods to combine this information with trial results [9]. This has been the dominant paradigm in the Bayesian analysis of RCT-based CEAs [10-14]. However, prior information on cost and typical effectiveness outcomes such as QALY is rarely available and if it is, it is often inappropriate to transfer to other settings [15,16]. This is because such outcomes are, to a large extent, affected by the specific settings in the jurisdiction in which they are measured (such as unit prices for medical resources). On the other hand, evidence on the aspects of the intervention that relate to the pathophysiology of the underlying health condition and the biologic impact of treatment, such as the effect size of treatment or rate of adverse events, are less affected by specific settings and are therefore more transferable [17]. This puts the investigator in a difficult situation for a RCT-based CEA as inference is made directly on cost and effectiveness using the observed sample, but evidence is available on some other aspects of treatment. One way to overcome this challenge is to create a parametric model to connect cost-effectiveness outcomes with parameters for which external evidence is available, and use Bayesian analysis, for example through Markov Chain Monte Carlo (MCMC) sampling techniques [18]. But such a model must connect several parameters through link functions, regression equations, and error terms. This involves a multitude of parametric assumptions and there is always the danger of model misspecification [19,20]. In addition, even with the advent of generic statistical software for Bayesian analysis, implementing such a model and comprehensive model diagnostics are not an easy undertaking. For an investigator using resampling methods for the CEA who wishes to incorporate external evidence in the analysis, this paradigm shift to parametric modeling can be a challenge.

In this proof-of-concept study, we propose and illustrate simple modifications of the bootstrap approach for RCT-based CEAs that enable Bayesian evidence synthesis. Our proposed method requires a parametric specification of the external evidence while avoiding parametric assumptions on the cost-effectiveness outcomes and their relation with the external evidence. The remainder of the paper is structured as follows: after outlining the context, a Bayesian interpretation of the bootstrap is presented. Next, the theory of the incorporation of external evidence into such sampling scheme is explained. A case study featuring a real-world RCT is used to demonstrate the applicability and face validity of the proposed method. A discussion section on the various aspects of the new method and its strengths and weaknesses compared to parametric approaches concludes the paper.

## Methods

### Context

Let *θ* = {*θ*_*i*_, *θ*_*e*_} be the set of parameters to be estimated from the data of a RCT and some external evidence. It consists of two subsets:  *θ*_*i*_, the parameter (s) of interest for which there is no external evidence, and *θ*_*e*_, some parameters for which external evidence is available. Typically, *θ*_*i*_ includes cost and effectiveness outcomes, and *θ*_*e*_ consists of some biological measures of treatment such as treatment effect. Let *D* represent the individual-level data of the current parallel-arm RCT, fully available to the investigator. We assume the population of interest for inference is the same as the population from which *D* is obtained, a fundamental assumption in any RCT-based CEA.

### Bayesian bootstrap

In a Bayesian context, the problem of inference on *θ* from a sample *D* can be conceptualized as incorporating some prior information with the information provided by the data to obtain a posterior distribution for *θ*:

(1) $$P\left(\theta |D\right)\propto \pi \left(\theta \right).P\left(D|\theta \right)$$

omitting a normalizing constant which is the function of *D*, but not *θ*. Here *π*(*θ*) is our prior distribution on *θ*, *P(D|θ)* is the likelihood of current data, and *P*(*θ*|*D*) is the posterior distribution having observed the trial data *D*. If prior and posterior distributions are from a parametric family indexed by a set of distribution parameters, then a fully parametric model can be used to draw inference on *P*(*θ*|*D*). However, one can perform such Bayesian inference non-parametrically: Rubin showed that if we assume a prior non-informative Dirichlet distribution for *D* itself (regardless of which parameter to estimate), then we can directly draw from *P*(*θ*|*D*) using a simple process called the Bayesian bootstrap [21]. In the Bayesian bootstrap of a dataset *D* consisting of *n* independent observations, a probability vector **P** = (*p*_1_, …, *p*_*n*_) is generated by randomly drawing from *Dirichlet*(*n*; 1, …, 1). The probability distribution that puts the mass of *p*_*i*_ on the *i*^*th*^ observation in *D* can be considered a random draw from the ‘distribution of the distribution’ that has generated *D*. Let D* represent a bootstrapped sample of *D* generated in this way, then according to the argument made above, θ*, the value of *θ* measured in this sample, is a random draw from *P*(*θ*|*D*) [21].

### Ordinary bootstrap as an approximation of the Bayesian bootstrap

The process of ordinary bootstrap can also be seen as generating a probability vector to the data, except only the probability vector is generated from the scaled multinomial distribution [22]. Such a process does not mathematically correspond to formal Bayesian inference. Nevertheless, the similarity in both the operation and results to the Bayesian bootstrap has led some investigators to interpret the ordinary bootstrap in a Bayesian way [23]. For example, the widely popular non-parametric imputation of missing data uses ordinary bootstrap as an approximate to the Bayesian bootstrap [22,24]. Indeed, it has already been shown that the ordinary and Bayesian bootstrap methods generate very similar results in non-parametric value of information analysis of RCT data [21]. Given this, for the rest of this work we use Bayesian and ordinary bootstraps interchangeably.

### CEA without the incorporation of external evidence

In a CEA in which we do not intend to incorporate any external evidence the quantity of interest for inference is *P*(*θ*|*D*). As described in the previous section, a sample from this quantity can be obtained using a simple resampling algorithm:

1 For *i* = 1,…,*M*, where *M* is the number of bootstraps:

a. Generate D*, a (Bayesian) bootstrap sample with bootstrapping performed within each arm of the trial.

b. Calculate θ* from D*.

2 Store the value of θ* and jump to 1.

This approach generates *M* random draws from the posterior distribution of *θ* having observed the RCT data. This is indeed the widely popular bootstrap method of RCT-based CEA [4]. An estimator for the ICER from the bootstrapped data can be obtained by calculating the ratio of the mean cost over mean effectiveness from the bootstrap samples [4]. Various methods can be used to construct a credible interval from the bootstrapped samples around this value [4,25]. These samples can also be used to present uncertainty in the form of a cost-effectiveness plane or cost-effectiveness acceptability curve (CEAC) [26].

### Incorporating external evidence

Let *D*_*e*_ be some external data providing evidence on *θ*_*e*_. While the external data is not fully available to the investigator, evidence is available most typically in the form of the external likelihood *P*(*D*_*e*_|*θ*_*e*_), for example, recovered from the reported maximum likelihood estimate and confidence bounds of treatment effect from a previously published study. We require *D* and *D*_*e*_ to be independent samples. This is a typical and fundamental assumption in evidence synthesis, for example in meta-analysis of treatment effect from multiple trials. By our definition of *θ*_*i*_ and *θ*_*e*_, we know that the external likelihood only provides information on *θ*_*e*_ (the information on *θ*_*i*_ is either not collected or is not reported by the investigators of the external study). As such, the external likelihood is a *marginal* likelihood for *θ*_*e*_ and hence is not a function of *θ*_*i*_. We also note that sometimes external evidence is obtained through a more subjective process, such as elicitation of expert opinion. In such cases, *D*_*e*_ becomes an abstract entity and *P*(*D*_*e*_|*θ*_*e*_) can be seen as a ‘weight’ function representing the degree of plausibility of *θ*_*e*_ against external knowledge.

In the presence of external data *D*_*e*_, the quantity of interest is *P*(*θ*|*D*, *D*_*e*_), which can be expanded, through three steps, as:

(2) $$\begin{array}{l} P\left(\;\theta_{i},\theta_{e}|D,D_{e}\right)\propto \pi \left(\;\theta_{i},\theta_{e}\right).P\left(D,D_{e}|\;\theta_{i},\theta_{e}\right)\propto \pi \left(\;\theta_{i},\theta_{e}\right).\\ P\left(D|\;\theta_{i},\theta_{e}\right).P\left(D_{e}|\;\theta_{i},\theta_{e}\right)\propto P\left(\theta |D\right).P\left(D_{e}|\theta_{e}\right) \end{array}$$

In the above derivations, in the first step we have applied the Bayes rule; the second step factorizes the likelihood given the independence of the external and current data; and the third step is based on the fact that the external data provides no information about *θ*_*i*_ (that is, *P*(*D*_*e*_| *θ*_*i*_, *θ*_*e*_) is not a function of *θ*_*i*_), so the likelihood term *P*(*D*_*e*_| *θ*_*i*_, *θ*_*e*_) is reduced to *P*(*D*_*e*_|*θ*_*e*_).

### Sampling from the posterior distribution

Suppose that a random sample can be generated from an ‘easy’ distribution *g*, but we are actually interested in obtaining a sample from a ‘difficult’ distribution *h*. How can we use the samples from *g* to obtain samples from *h*? Two popular methods for converting samples from *g* to *h* are rejection sampling [27] and importance sampling [28]; both are based on applying weights proportional to density ratio *h*/g to each observation from *g*. In the present context, *g* = *P*(*θ*|*D*) and *h* = *P*(*θ*|*D*, *D*_*e*_); the weights are, according to (Equation 2), proportional to *P*(*D*_*e*_|*θ*_*e*_). That is, to obtain samples from *P*(*θ*|*D*, *D*_*e*_), each θ* as a sample from *P*(*θ*|*D*), obtained through bootstrapping, needs to be weighted by $P\left(D_{e}|\theta_{e}^{*}\right)$. To operationalize this, we propose two approaches based on rejection and importance sampling schemes. The reader can refer to Smith and Gelfand for an elegant elaboration on these two sampling schemes (along with the derivations) [27].

### Rejection sampling

In this scheme, each D*, the entire bootstrap sample of the RCT data, is accepted by a probability that is proportional to $P\left(D_{e}|\theta_{e}^{*}\right)$, the weight of $\theta_{e}^{*}$ obtained from D*. This results in the following algorithm:

1 For *i* = 1,…,*M*, where *M* is the desired size of the sample:

a. Generate D*, a (Bayesian) bootstrap sample of *D*, with bootstrapping performed separately within each arm of the trial.

b. Calculate the parameters $\theta^{*}=\left\{\theta_{i}^{*},\theta_{e}^{*}\right\}$ from this sample.

c. Calculate $P^{*}=P\left(D_{e}|\theta_{e}^{*}\right)$, the weight of $\theta_{e}^{*}$ according to external evidence.

d. Randomly draw *u* from a uniform distribution in the interval [0,1]. If *u* > P* , then ignore the bootstrap sample and jump to step a.

2 Store the value of θ* and jump to 1.

This approach generates *M* random draws from the posterior distribution of *θ* having observed the RCT data and the external evidence. All the subsequent steps of the CEA, such as calculating the average cost and effectiveness outcomes, interval estimations, and drawing the cost-effectiveness plane and the CEAC, remain unchanged. Of note, this algorithm requires that P* be valid probabilities bounded between 0 and 1. As such, the external likelihood should be scaled (e.g., divided by $\max_{\theta_{e}}\left[P\left(D_{e}\left|\theta_{e}\right)\right)\right]$).

### Importance sampling

As an alternative to probabilistically accepting or rejecting bootstrap samples one can assign the weights directly to each bootstrap sample [27]. That is, one proceeds by obtaining a desired number of bootstraps, calculating $\theta_{e}^{*}$ in each sample, and assigning a weight proportional to $P\left(D_{e}|\theta_{e}^{*}\right)$ to each bootstrap. All subsequent calculations require incorporating such weights (for example, ICER will be the ratio of the weighted mean of costs over the weighted mean of effectiveness).

### Regularity conditions

Fundamental to the proposed sampling scheme is that the joint likelihood of *D* and *D*_*e*_ can be factorized into two independent likelihoods. The onus is on the investigator to ensure this condition is satisfied with at least a good approximation. This can be context-specific. A few scenarios that violate this assumption are when *D* and *D*_*e*_ have overlapping samples, when *D*_*e*_ is an estimate from a meta-analysis of studies that included the current study *D*, or when *D*_*e*_ represents experts’ opinion about treatment effect if their opinion is already influenced by the results of the current study (the hindsight bias [29]).

In addition, the general regularity conditions required for the rejection and importance samplings should hold [27]. Particularly, since *P*(*θ*|*D*) is most often continuous (or for the regular bootstrap it takes many discrete values), the external likelihood *P*(*D*_*e*_|*θ*), should also be continuous, otherwise the chance of samples from *P*(*θ*|*D*) hitting non-zero areas of *P*(*D*_*e*_|*θ*) will be infinitely small. Next, *θ*_*e*_ should be identifiable (unique) within each D*. This assumption holds for the most typical form of external evidence such as rates or measures of relative risk [30]. Further, *P*(*D*_*e*_|*θ*) should be bounded. If *P*(*D*_*e*_|*θ*) has an infinite maximum, for example, if it is proportional to the density function of a beta distribution with either of its parameters being less than one the proposed sampling schemes might fail. Such distributions are, however, mainly used as non-informative priors and seldom represent external evidence in realistic scenarios. On the other hand, mixed-type distributions such as the so called lump-and-smear priors that put point mass on the value of the parameter consistent with the null hypothesis ( [31] page 161), have unbounded density functions and cannot readily be used in the proposed sampling methods.

We used data from a real-world RCT to show the practical aspects of implementing the proposed algorithms. Ethics approval was obtained from the Ottawa Hospital Research Ethics Board (#2002623-01H) and Vancouver Coastal Health Authority (#C03-0275).

## Results

### An illustrative example

This case study is to demonstrate the operational aspects of implementing the algorithm and is not intended to be a practice in comprehensive evidence synthesis to inform policy.

The case study is based on the OPTIMAL trial, a multicenter study evaluating the benefits of combination pharmacological therapy in preventing respiratory exacerbations in patients with chornic, obstructive pulmonary disease (COPD) [32,33]. Pharmacological treatment of COPD, typically with inhaled medications, is often required to keep the symptoms under control and reduce the risk of exacerbations. Sometimes patients receive combinations of treatments of different classes in an attempt to bring the disease under control. However, there is a lack of evidence on whether such combination therapies are effective. The OPTIMAL trial was designed to estimate the comparative efficacy and cost-effectiveness of single and combination therapies in COPD. It included 449 patients randomized into three treatment groups: T1: monotherapy with an inhaled anticholinergic (tiotropium, N = 156); T2: double therapy with an inhaled anticholinergic plus an inhaled beta-agonist (tiotropium + salmeterol, N = 148); and T3: triple therapy with an inhaled anticholinergic, an inhaled beta-agonist, and an inhaled corticosteroid (tiotropium + fluticasone + salmeterol, N = 145). The primary outcome measure of the RCT was the proportion of patients who experienced at least one respiratory exacerbation by the end of the follow-up period (52 weeks). This outcome was not significantly different across the three arms: the odds ratio (OR) for the risk of having at least one exacerbation by the end of the follow-up period was 1.03 (95% CI, 0.63 to 1.67) for T2 versus T1 and 0.84 (95%CI, 0.47 to 1.49) for T3 versus T1 (lower OR indicates a better outcome). Because the T2 arm in the OPTIMAL trial was dominated (was associated with higher costs and worse effectiveness outcomes) in the original CEA, and for the sake of brevity, in this case study we restrict the analysis to a comparison between T3 and T1.

Details of the original CEA are reported elsewhere [34]. Data on both resource use and quality of life were collected at individual level during the trial, which was used to carry out the CEA. The main outcome of the CEA was the incremental costs per QALY gained for T3 versus T1 (that is, the difference in mean costs over the difference in mean QALYs). Since individual level resource use and effectiveness outcomes were available, the CEA was based on the direct inference on their distribution. No external information was incorporated in the analysis in the original CEA.

### External evidence

The set of parameters with external evidence in this analysis (*θ*_*e*_) consists of one quantity: the logarithm of rate ratio (RR) of exacerbations between T3 and T1 (denoted by *θ*_*T*3,*T*1_) within the follow-up period. We used a formal process for evidence synthesis by performing a MEDLINE search for all clinical trials as well as systematic reviews on the treatment effect of combination pharmacotherapies for COPD. In synthesizing evidence, we assumed a ‘class effect’ for the study medications, in line with conventional wisdom and several pharmacoepidemiology studies evaluating such medications in COPD [35-37]. The most relevant source of evidence on the effect size of T3 versus T1 was from a RCT on comparing budesonide (in the same class as fluticasone) and formoterol added to tiotropium versus tiotropium alone in COPD patients [38]. This study reported a RR of 0.38 (95% CI 0.25 to 0.57). The evidence was parameterized by using normal likelihoods on the log-RR scale. When transferring evidence form one setting to another it is important to consider the likely presence of between-study variation (due to difference in inclusion criteria, treatment protocol, measurements, and so on) [39]. Because only one study on this comparison was at hand, no estimate for between-study variation could be obtained. As such, we use the estimated between-study variance of 0.01783 from the multiple-treatment comparison of COPD treatments (personal communication with the author K Thorlund) [35]. This results in the external evidence being associated with a RR of 0.38(95% CI 0.24 to 0.59), thus:

(3) $$\log\_\mathrm{RR}\sim \mathrm{Normal}\left(\mu ,\sigma \right),\mu =-0.968,\sigma =0.246$$

with *μ* and *σ* corresponding to the mean and standard deviation of the normal distribution. We note that the uncertainty around the log-RR from external evidence, represented by the above probability distribution, stems from two sources: the finite sample of the external study, and our assumption on between-study variability. Overall, the RR representing external evidence is much more in favor of combination therapy than the RR observed in the OPTIMAL trial. As such, we *a priori* expect that the incorporation of external evidence shall improve the cost-effectiveness outcomes in favor of T3.

Putting all these together, the external evidence can be parameterized as:

(4) $$P\left(D_{e}|\theta \right)\propto e^{-\frac{{\left(\theta_{T3,T1}-\mu \right)}^{2}}{2\sigma^{2}}}\propto e^{-\frac{{\left(\theta_{T3,T1}+0.968\right)}^{2}}{0.121}}$$

a normal likelihood function representing our knowledge on treatment effect. The original algorithm for the CEA can now be updated to incorporate the external evidence as follows (using the rejection sampling scheme):

1 For *i* = 1,2,…,*M*.

a. Generate D^*^, a (Bayesian) bootstrap sample within each of the three arms of the RCT.

b. Impute the missing values in costs, utilities, and exacerbations in D^*^.

c. Calculate $\;\theta_{T3,T1}^{*}$, the *log*(*RR*) of exacerbation during the follow-up period for T3 vs. T1 from the bootstrapped sample.

d. Calculate $\;P=P\left(\theta_{T3,T1}^{*}\right)$ using the distribution constructed for the external evidence.

e. Randomly draw *u* from a uniform distribution in the interval [0,1]. If *u* >*P*, then ignore the bootstrapped sample and jump to step a.

f. . Calculate mean costs, exacerbations, and QALYs for each arm from D^*^.

2 Store the average values for costs, exacerbation rates, and QALYs; then jump to 1.

The simulation was stopped after 10,000 accepted bootstraps for the rejection sampling method incorporating the external evidence were generated. To obtain the results using the importance sampling method, we used the same set of bootstraps generated in the above algorithm, including all the accepted and rejected bootstraps.

In addition to the ICER, we also reported the expected values of the cost and health outcomes for each trial arm, and also plotted the CEAC, without and with the incorporation of the external evidence. The CEAC between two treatments is the probability that a treatment is cost-effective compared to another at a given value of the decision-maker’s willingness-to-pay (*λ*) for one unit of the health outcome [26]. The statistical code for this case study is provided in Additional file 1.

### Results of the case study

Table 1 presents the expected value costs and QALYs for the T1 and T3 arms of the OPTIMAL trial without and with the incorporation of the external evidence. The Bayesian and ordinary bootstraps generated very similar results (Table 1). Similarly, results from the rejection and importance sampling methods were very similar (results not shown).

**Table 1:** Outcomes of the OPTIMAL CEA without and with the incorporation of external evidence*

		**T1**	**T3**	**Difference (T3 – T1)**	**ICER**
No external evidence					
*Bayesian bootstrap*	Costs	2649 (466)	4074 (547)	1425 (721)	250,329
	QALY	0.7071 (0.0075)	0.7128 (0.0093)	0.0057 (0.0087)	
*Ordinary bootstrap*	Costs	2650 (467)	4077 (551)	1427 (721)	251,171
	QALY	0.7071 (0.0075)	0.7128 (0.0093)	0.0057 (0.0087)	
With external evidence					
*Bayesian bootstrap*	Costs	2753 (492)	3959 (510)	1205 (709)	121,260
	QALY	0.7053 (0.0074)	0.7152 (0.0092)	0.0099 (0.0085)	
*Ordinary bootstrap*	Costs	2742 (477)	3966 (536)	1225 (709)	126,387
	QALY	0.7054 (0.0074)	0.7151 (0.0092)	0.0098 (0.0084)	

*Results are mean (*standard deviation*).

*ICER, incremental cost-effectiveness ratio; QALY, quality-adjusted life year*.

As this table demonstrates, the incorporation of external evidence shifted the outcomes of the T3 arm in the favorable direction (lower costs and higher QALYs), and shifted the outcomes of the T1 arm in the opposite direction. This is an expected finding given the strong evidence in favor of T3 for the effect size of T3 versus T1 from the external source.

**Figure 1:**
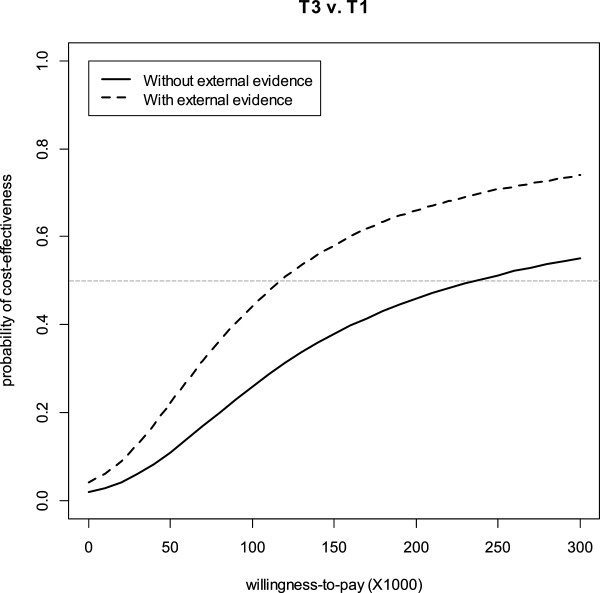
**Cost**-**effectiveness acceptability curve (CEAC) without and with the incorporation of external evidence.** The horizontal grey line represents the 50% threshold on probability of cost-effectiveness.

The impact of incorporating external evidence is more evident on the ICER. The ICER of T3 versus T1 decreased by 52% after the incorporation of external evidence. Again, this is reflective of the fact that external evidence is more in favor of T3 than the likelihood (RCT data) is.Figure 1 presents the results of incorporating external evidence on the CEAC (using the Bayesian bootstrap). The incorporation of external evidence increased the probability of cost-effectiveness for T3, especially with higher willingness-to-pay (λ) values. Without the incorporation of external evidence, the probability of T3 being cost-effective compared to T1 reach the 50% threshold at λ values greater than $240,000/QALY, while the incorporation of the external evidence moved this threshold to $115,000/QALY.

## Discussion

Contemporarily, when an economic evaluation is conducted alongside a single RCT, the practice of evidence synthesis is not an integral part of the analysis. In our opinion, this is partly because parametric Bayesian modeling, the hitherto only available method, results in problem-specific and complex statistical models. In this work we propose simple and intuitive algorithms for the incorporation of external evidence in RCT-based CEAs that use bootstrapping to draw inference. Rejection and importance samplings which form the basis of the proposed method are popular paradigms in which sampling from a ‘difficult’ distribution is replaced by sampling from a proposal (or instrumental) distribution [40]. Here, sampling from *P*(*θ*|*D*, *D*_*e*_)  is performed via *P*(*θ*|*D*), and the latter can easily be sampled through (Bayesian) bootstrapping.

In synthesizing evidence for RCT-based CEAs, a carefully crafted parametric model with comprehensive analysis of model convergence and sensitivity of results to parametric assumptions has indisputable strengths over resampling approaches, including the higher computational efficiency of MCMC or likelihood-based methods and the ability to synthesize and propagate all evidence in a single analytical framework [41,42]. Nevertheless, important advantages make the proposed resampling methods a competitive option. The proposed methods are intuitive and easy extensions of the popular bootstrap method of RCT-based CEAs; they do not require specialist software and in-depth content expertise for implementation. In addition to such practical advantages, the proposed resampling methods connect the parameters for which external evidence is available to the cost and effectiveness outcomes without an explicit model, which is a requirement in parametric Bayesian approaches.

Our paper provides a conceptual framework and further research into theory, as well as practical issues in using this method, should follow. The apparent simplicity of the bootstrap may conceal the assumptions being made, especially with small datasets [21,43]. Furthermore, if the external evidence and RCT data substantially differ on the information they provide for the evidence (that is, that the prior and data are in conflict) [44], or when there are multiple parameters for which external evidence is available, then the sampling methods will become inefficient.

Further research is needed to improve sampling efficiency and to incorporate external evidence in other paradigms such as cluster or crossover RCTs. Importantly, the theoretical construct of the proposed method does not necessarily restrict it to RCT-based CEAs. A similar concept can be used to reconcile evaluations based on observational data with external evidence. This will inevitably invoke questions about the applicability of different metrics of the effect size in non-randomized studies (for example, average treatment effect versus average treatment effect for the treated), and the validity of the bootstrap as the sampling method (for example, in a propensity-score-matched cohort). In addition, further empirical research is required to evaluate the real-world applicability and feasibility of the method and to demonstrate its comparative performance against conventional methods of evidence synthesis (for example, parametric Bayesian analysis using MCMC).

This paper deliberately stays away from the debate on whether to incorporate external evidence for a given situation an d focuses on the ‘how to’ question. The ‘whether to’ question is context-specific and great care is required for the sensible use of external evidence in each setting. For the case study, for example, the substantial discrepancy in the results between the external and current RCTs (with regard to the efficacy of triple therapy versus monotherapy) should more than anything generate misgivings about the suitability of borrowing evidence from that external source. However, the case study was undertaken as a step in the direction of proof of concept, applicability, and face validity of the proposed methods. This is not a withdrawal from the deep considerations required for sensible evidence synthesis.

## Conclusions

Faced with the escalating costs of RCTs and the requirement by many decision-making bodies for formal economic evaluation of emerging health technologies, trialists and health economists are hard-pressed to generate as much relevant information for policymakers as possible. As such, and despite criticisms, it appears that RCT-based CEAs are here to stay. The incorporation of external evidence helps optimize adoption decisions. Aside from their theoretical contribution, if their real-world applicability is proven the proposed methods can provide the large camp of analysts using bootstrap for RCT-based CEAs with a statistically sound, easily implementable tool for such purpose.

## Abbreviations

CEA: Cost-effectiveness analysis; CEAC: Cost-effectiveness acceptability curve; COPD: Chronic obstructive pulmonary disease; ICER: Incremental cost-effectiveness ratio; MCMC: Markov chain Monte Carlo; OR: Odds ratio; RCT: Randomized controlled trial; RR: Rate ratio; QALY: Quality-adjusted life year.

## Competing interests

The authors declare that they have no competing interests.

## Authors’ contributions

This work was part of MS’ PhD research. MS developed the research question and the methodology. MS and SB designed the case study. CM and SA helped with the acquisition of the data and provided content advice for the case study. MS performed the computer simulations. MS and SB developed the first draft of the manuscript. All authors critically revised the manuscript and approved the final version.

## Supplementary Material

Additional file 1 **File name: R code.r.** Description: This is the R code used for the case study. Click here for file
